# Polymer Composite Thermoforming: Ultrasonic-Assisted Optimization for Enhanced Adhesive Performance in Automotive Interior Components

**DOI:** 10.3390/polym16010052

**Published:** 2023-12-22

**Authors:** Liufei Yue, Weiguo Yao, Fei Teng, Yanchao Zhu, Zengxia Zhao, Ce Liang, Lijuan Zhu

**Affiliations:** 1 Key Laboratory of Automobile Materials, Ministry of Education, College of Materials Science and Engineering, Jilin University, Changchun 130025, China; yuelf21@mails.jlu.edu.cn (L.Y.);; 2 College of Mechanical and Automotive Engineering, Changchun University, Changchun 130025, China; 3 College of Chemistry, Jilin University, Changchun 130025, China

**Keywords:** epoxy resins, ultrasonic-assisted forming, thermo-mechanical coupling, adhesion

## Abstract

Dual-component epoxy resins are widely used for bonding different materials in automotive interior processing. However, due to the complexity and variability of automotive interior parts, uneven temperature distribution on curved surfaces during the thermoforming process can lead to uneven thermal stress distribution, damaging the interior components. This study focuses on addressing the damage issues caused by uneven thermal stress distribution during the thermoforming of automotive interior components. By monitoring the temperature and strain on the adhesive surface of the interior components during processing, using sensors and combining the readings with a finite element simulation, damage to the adhesive during processing was simulated. Based on this, a segmented thermoforming method for the model surface was employed, but it was found that this method did not significantly reduce the level of damage to the adhesive during application. Building upon the segmented simulation, significant results were achieved by applying temperature modulation at a certain frequency to adjust the damage of the interior components during processing. The techniques used in this study successfully reduced the unevenness of the adhesive surface temperature, improved the performance of the adhesive during application through segmented optimization and the application of ultrasound-assisted techniques, and markedly reduced the manufacturing process’s energy consumption.

## 1. Introduction

In the current field of automotive interior component manufacturing, challenges such as temperature unevenness, stress concentrations, and production costs significantly impact the quality and performance of interior components. Despite existing research on adhesive damage, there remains a gap in addressing these issues. This study aims to fill this gap by optimizing adhesive performance and mitigating the damage caused by temperature non-uniformity by applying zone-specific hot-press molding and ultrasound-assisted temperature modulation techniques [1,2].

To better comprehend the motivation and objectives of this research, it is essential to emphasize the significance of these issues in the realm of automotive interior manufacturing. Resolving temperature non-uniformity and adhesive damage issues enhances the performance and durability of interior components and contributes to cost reduction, thereby driving progress in the entire industry [3]. The existing literature on adhesive damage to automotive interior components provides valuable background information. However, the current literature primarily focuses on observing and analyzing damage, with fewer methods proposed for mitigating and preventing such damage. This study seeks to provide practical solutions for addressing the adhesive damage induced by temperature non-uniformity by adopting ultrasound-assisted technology and zone-specific simulations [4,5].

Ozawa’s [6] research focused on the influence of heating rate on the thermal weight curve of polymers and proposed a simple and user-friendly method for extracting kinetic parameters from the thermal weight curve. Hillerborg [7] et al. introduced fracture mechanics into finite element analysis and validated this behavior by assuming a situation where stress is acting on a narrow crack, confirming the fracture energy absorption’s expression via the energy balance method. Benzeggagh [8] et al. evaluated initiation and delamination growth in epoxy resin materials under different modes through static loading, describing the performance of these modes via the total critical strain energy release rate and total fracture resistance. Vyazovkin [9] proposed a nonlinear algorithm to improve the accuracy of activation energy assessment by integrating numerous isothermal methods. This advanced isothermal method reliably estimates the activation energy needed when the thermal effects of a reaction cause the sample temperature to deviate from the predetermined heating program. Vyazovkin [10] further developed a computational method applicable to arbitrary scenarios of temperature changes caused by the thermal effects of reaction systems and provided detailed information regarding numerical algorithms. Sbirrazzuoli [11] applied differential scanning calorimetry (DSC) under isothermal and non-isothermal conditions and introduced dynamic rheology and temperature-modulated DSC to study the properties of epoxy resins and amine curing agents. Hahn [12] studied thermal expansion in the automotive industry, curing shrinkage, the relative motion between bonded materials, and the final deformation of single-component epoxy adhesives through experiments and the use of numerical methods. Willam [13] introduced the thickness interface model and studied the degradation of the interfacial transition zone in laminated materials, as caused by thermal and mechanical damage. Vyazovkin [14] studied the curing kinetics of epoxy resin materials using an isothermal method and achieved good results. Sergey Vyazovkin [15] et al. discussed the problems faced when using various kinetic methods and provided advice on analyzing and interpreting the results for non-experts. Priesnitz [16] explored the application of adhesives in the automotive field, established models, and analyzed the effects of thermodynamic and chemical volume changes, curing processes, solid–liquid phase transitions, and stress relaxation on adhesive applications. Burbulla [17] established a material damage model based on continuum mechanics and damage mechanics theory, which can be used to predict the complex mechanical behavior of damaged high-strength adhesives under shear and tensile loads. Schiel [18] conducted a correlation study of different thermal analysis techniques for epoxy resin materials and gained a deep understanding of the development of mechanical properties during the curing process. Apalak [19] investigated the wide application of adhesives in connecting similar or dissimilar materials in various engineering fields. He analyzed the fracture behavior caused by differing layer structures and mismatched coefficients of thermal expansion when applying adhesives. Simões [20] studied the influence of temperature stress on the connectivity performance of multi-layered materials during adhesive curing. By establishing a finite element analysis model, he analyzed the impact of adhesive curing reactions on the connectivity of multi-layered materials from multiple perspectives.

Liang [21] et al. systematically analyzed the influence of applied pressure on the epoxy resin curing process and established a model to explore the effects of applied pressure during the curing process. Zhao [22] et al. initiated the study of temperature effects when applying epoxy resins in the automotive field. Nester [23] et al. focused on processing defects in epoxy resin materials that were caused by external processing conditions and proposed various improvement measures. Safaei [24] et al. proposed a new technique for measuring the fracture toughness of brittle adhesives (Type II critical strain energy release rate), providing a new perspective for finite element studies of epoxy resin curing reactions.

Yao [25] et al. studied the damage caused to the adhesive layer of automotive interior components, specifically the dashboard, by applying different displacements in various directions during manufacturing. Their research provided theoretical guidance for reducing adhesive damage during the processing of interior components. However, they did not investigate the specific processing techniques needed to mitigate adhesive damage during manufacturing. Li et al. [26] used the cohesive zone model to discover damage to adhesives during the manufacturing process of automotive interior components. They found that the damage found in the adhesive layer between the PP skeleton and non-woven fabric was more significant than the damage found in the polyurethane skin and adhesive. However, they did not provide specific methods to reduce adhesive damage.

In the current research, although relevant studies have been published on adhesive damage to automotive interior components, unresolved issues and novel research directions still require further exploration. The existing literature predominantly focuses on the observation and analysis of damage, with relatively fewer methods proposed for mitigating and preventing such damage. Research on the adhesive damage induced by temperature non-uniformity in highly curved areas remains relatively limited. Therefore, this study aims to address these gaps in the research field, specifically focusing on optimizing adhesive performance during the automotive interior manufacturing process. This is achieved by applying zone-specific hot-press molding and ultrasound-assisted technology, which involves controlling temperature and pressure with the use of a specific frequency. The study aims to delve into damage formation mechanisms through a combination of simulation and experimentation, providing practical and viable solutions to mitigate the damage caused by temperature non-uniformity. Ultimately, this research aims to propel the manufacturing technology of automotive interior components forward and reduce manufacturing industry energy consumption [1,2,3,4,5].

## 2. Materials and Models

### 2.1. Materials and Model Parameters

#### 2.1.1. Material Properties

The same material parameters were employed in both the experiments and the simulations to ensure the universality of experimental and simulation results. The components of the automotive interior parts primarily included polyurethane skin (provided by Wanhua Chemical, Yantai City, China), a two-component epoxy resin adhesive (H.B. Fuller Adhesive Co., Ltd., Shenzhen City, China.) with a glass transition temperature of 65 ± 5 °C, and polyurethane non-woven fabric (provided by Wanhua Chemical, Yantai City, China) [25,26].

The processing of automotive interior parts generally involves two steps. In the first step, the two-component epoxy resin and amine curing agent are uniformly applied in a 1:1 ratio on polyurethane non-woven fabric. The adhesive is cured when at the curing temperature, reaching the point where the adhesive transforms into an elastic body. At this stage, the second step is executed by placing both components into an automatic molding device’s upper and lower molds for automotive interior part coating. This study specifically focuses on the second step, involving the process of assembling the interior components with the polyurethane skin when the adhesive transforms into an elastic body.

The polyurethane in the non-woven fabric and the polyurethane skin contains urethane groups that are compatible with isocyanate. Therefore, these materials are highly suitable for interior part applications. The parameters for the polyurethane skin component (provided by Wanhua Chemical), the mesh fabric parameters (provided by Wanhua Chemical), and the two-component epoxy resin adhesive Araldite ^®^ 2015 (Fuller (China) Adhesive Co., Ltd., Shenzhen City, China.) are detailed in Table 1 and Table 2, encompassing the mechanical and temperature-related aspects of the three-layer material.

#### 2.1.2. Relevant Parameters

Table 1 and Table 2 not only provide the fundamental parameters of density, Young’s modulus, Poisson’s ratio, and other parameters for the polyurethane skin, mesh fabric, and adhesive, but also incorporate the thermal conductivity, coefficient of thermal expansion, and specific heat values for polyurethane skin and mesh fabric. These parameters form the foundation for simulating the mechanical performance and heat transfer of multilayer materials. Regarding the adhesive, in addition to the aforementioned common parameters, the fracture energy and shear energy are included, establishing the groundwork for studying the adhesive’s damage conditions. In addition to assigning material properties to the multilayer materials in Table 1 and Table 2, a temperature of 70 °C was applied to the surface of the polyurethane skin, and a pressure of 0.1 MPa was exerted on its surface while maintaining the entire stamping process in a vacuum environment. The application of 70 °C was chosen due to the glass transition temperature of the adhesive being 65 ± 5 °C, preparing the cured adhesive to reach an elastic state for its subsequent bonding with the polyurethane skin. Simultaneously applying 0.1 MPa of pressure ensured a more robust bonding between the polyurethane skin and the adhesive.

#### 2.1.3. Model Description, Simplification, and Mesh Partitioning

The model is based on the cohesive zone model and comprises three layers of materials. The surface of the model consists of a polyurethane skin, the middle layer is a two-component epoxy resin adhesive, and the bottom layer comprises a 3-D mesh fabric. For computational efficiency and because of the critical nature of the problem, the model incorporates the 3-D mesh fabric and polyurethane skin based on the parameters provided in Table 1 and Table 2. The cohesive zone model (CZM) is employed to connect the polyurethane skin and 3-D mesh fabric. Given the thinness of the adhesive layer (0.1 mm), after fixing the 3-D mesh fabric and polyurethane skin according to the specified parameters, the adhesive layer’s thickness is simplified to 0. Additionally, the molds used in the processing of the interior components are simplified, allowing the direct imposition of boundary conditions on the model’s surface.

This streamlined geometric representation facilitates more effective numerical analysis, contributing to a reduction in computational complexity while maintaining an accurate description of the problem’s essence. The polyurethane skin and mesh fabric are meshed using hexahedral elements in a structural manner, employing the implicit form of coupled temperature displacement for meshing. The aforementioned analysis step has been chosen to facilitate the model by simultaneously meeting the specified loading time, temperature, and pressure requirements during the simulation calculations. Due to the unique properties of the adhesive, meshing is done using hexahedral elements in a sweeping manner, employing the cohesive family of elements for meshing.

#### 2.1.4. Model Boundary Conditions and Analysis Step

The imposition of model boundary conditions simulates the actual stamping process, employing ABAQUS simulation software with a coupled temp-displacement analysis step. The entire simulation of the stamping process lasts for 10 s. The selection of the coupled temp-displacement analysis step is based on its ability to simultaneously accommodate the heating and pressurizing processes of the interior parts during simulation while also defining the time needed for the entire model’s heating and pressurizing, meeting the simulation requirements of the actual interior part-stamping process. The specific boundary conditions for the model are applied as follows: a fixing treatment is performed on the model’s bottom surface, maintaining fixed constraints for all six degrees of freedom, i.e., U1 = U2 = U3 = UR1 = UR2 = UR3 = 0. Simultaneously, the entire model commences with stamping at room temperature, i.e., 25 °C, and the temperature during the stamping process is applied to the model’s upper surface in the form of boundary conditions, eventually reaching 70 °C. Regarding the temperature application, during the simulation of the interior component manufacturing process, the ambient temperature is maintained at 25 °C. In the heating process, considering that the temperature cannot immediately reach 70 °C, it is gradually increased from room temperature to 70 °C.

**Figure 1:**
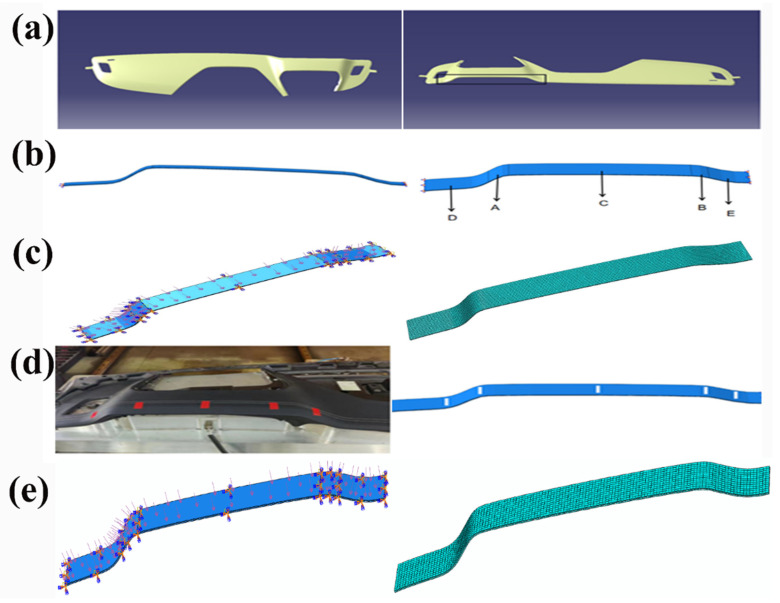
(**a**) Car dashboard and area selected for simulation within the black box on the dashboard; (**b**) local three-dimensional model of the dashboard and diagram of the model’s region partitioning; (**c**) boundary conditions of the model and applied pressure and the mesh partitioning of the model; (**d**) diagram of the area of the dashboard used for the specimen, marked in red, and a schematic diagram of the model’s region-partitioning marking; (**e**) boundary conditions of the model along the thickness direction, with the mesh division of the model along the thickness direction.

Stamping pressure is applied to the model’s upper surface, in the form of pressure with the parameter set at 0.1 MPa. The layers of the model are in face-to-face contact with each other, and the contact properties between the faces employ the cohesive behavior of the contact material’s properties. The molds used in the processing of interior components are simplified, and thus, the boundary conditions are directly applied to the model’s surface. This streamlined geometric representation facilitates more effective numerical analysis, reducing the computational complexity while maintaining an accurate description of the problem’s essence. Figure 1 shows the display of various parts of the model, including examples of the model, mesh division, boundary conditions, and comparison with the actual object.

### 2.2. Experimental Setup

#### 2.2.1. Experimental Design

The experiment employs fully automated interior trim molding technology. The cured semi-finished product, with a PP framework bonded to the mesh fabric and with simultaneously cured double-component epoxy adhesive on the mesh fabric, is placed on the lower mold of the covering device. The polyurethane skin material is placed on the upper mold of the covering device. Before the molds are closed, the adhesive is heated using a hot air pipe until it reaches the glass transition temperature. The molds are then closed and, during the molding process, the upper surface of the interior trim is heated and pressurized through the molds. This ensures that the heating temperature of the polyurethane surface of the interior trim reaches 70 °C and the surface pressure reaches 0.1 MPa. During molding, sensors record changes in the temperature, stress, strain, and displacement of the adhesive in the interior trim. Subsequently, a detailed analysis of the acquired data is performed to elucidate the changes in the adhesive layer during the processing of the interior trim.

#### 2.2.2. Measurement Techniques

**Figure 2:**
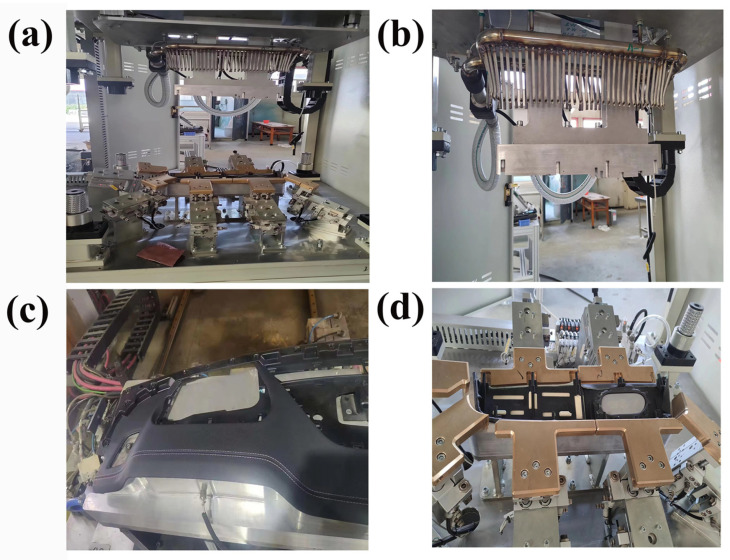
(**a**) Interior of the fully automatic wrapping device; (**b**) the hot air duct; (**c**) the workpiece; (**d**) the workpiece in the mold clamping device.

The interior trim molding process uses a fully automated covering and molding device, as shown in Figure 2.

Figure 2 shows an overall schematic of the interior trim processing device, including the heating equipment used for interior trim processing, the upper and lower molds, and a sensor device placed on the upper surface of the molds. This sensor records the temperature, stress, strain, and displacement changes during the covering and molding of the interior trim. Figure 2b illustrates the heating device for the adhesive. The dual-component epoxy adhesive is heated using a hot air pipe, ensuring that the adhesive’s temperature reaches the glass transition temperature. Figure 2c displays the finished product of the entire interior trim molding process, and Figure 2d depicts the covering mold for the interior trim.

### 2.3. CZM Principal Structure Model

#### 2.3.1. Basic Principles of the CZM Model

**Figure 3:**
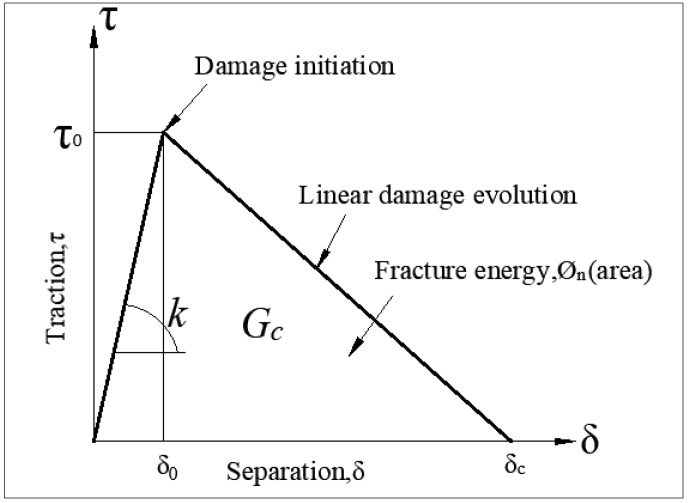
Bilinear intrinsic structure model.

Currently, the two commonly used models for material damage in the field are linear elastic fracture mechanics (LEFM) and the cohesive zone model (CZM). The LEFM model requires prior knowledge of the crack’s damage path, whereas the CZM model does not require prior knowledge of the crack initiation damage. Therefore, the advantage of the CZM model lies in its ability to handle damage without prior information about the crack [25]. As external forces and temperature must be applied simultaneously to the material in the experiments, it is crucial to consider the coupling of temperature and external loading. This paper uses a bilinear principal structure equation to model cohesion (as shown in Figure 3), where $G_{c}$ is the fracture toughness, $\tau_{0}$ is the material strength, and *K* is the initial stiffness of the material [27,28].

(1) $${\left\{\frac{\left\langle t_{n}\right\rangle }{t_{n}^{max}}\right\}}^{2}+{\left\{\frac{t_{s}}{t_{s}^{max}}\right\}}^{2}+{\left\{\frac{t_{t}}{t_{t}^{max}}\right\}}^{2}=1$$

Here, $t_{n}$ is the normal nominal stress of the cohesive element, $t_{n}^{max}\;is$ the normal critical stress, $t_{s},\;t_{t}$ is the nominal stress in both tangential directions, and $t_{s}^{max}$ $t_{t}^{max}$ is the tangential critical stress.

(2) $$G_{Ic}+\left(G_{IIc}-G_{Ic}\right){\left(\frac{G_{s}}{G_{T}}\right)}^{\eta }=G_{Tc}$$

Here, $G_{Ic}$ is the normal fracture energy, $G_{s}$ is the sum of type II and type III fracture energy values, and $G_{T}$ is the sum of the three fracture energies, where $G_{IIc}$= $G_{IIIc}$ and η is the damage factor, which is generally between 0.5 and 2.0.

#### 2.3.2. Theoretical Foundation for the Mixed Loading of Temperature and Stress

Since the process requires two steps, the activation of the adhesive layer and the application of pressure to form the laminate, the actual loading process is a mixed-mode loading process; thus, a thermodynamically uniform damage model was chosen for loading. This procedure first determines the degree of CE mixing between pure modes I and II by defining *β* (mode mixing ratio) and then defining the cohesion law based on the actual mode mixing ratio, as follows:

(3) $$\delta_{m}^{2}={\left\langle \delta_{n}\right\rangle }^{2}+\delta_{sh}^{2}$$

(4) $$\beta =\frac{\delta_{sh}}{\delta_{m}}$$

(5) $$B=\frac{G_{\mathrm{shear}\;}}{G_{T}}=\frac{\beta^{2}}{1+2\beta^{2}-2\beta }=\frac{\delta_{sh}^{2}}{\delta_{m}^{2}}$$

(6) $$\delta_{0_{m}}^{2}={\left(\delta_{0_{n}}\right)}^{2}+\left[{\left(\delta_{0_{sh}}\right)}^{2}-{\left(\delta_{0_{n}}\right)}^{2}\right]B^{\eta }$$

(7) $$\delta_{f_{m}}=\frac{\delta_{0_{n}}\delta_{f_{n}}+\left(\delta_{0_{sh}}\delta_{f_{sh}}-\delta_{0_{n}}\delta_{f_{n}}\right)B^{\eta }}{\delta_{0_{m}}}$$

(8) $$\tau_{0_{m}}=K\delta_{0_{m}}$$

(9) $$G_{c_{m}}=G_{c_{I}}+\left(G_{c_{II}}-G_{c_{III}}\right)B^{\eta }$$

(10) $$d=\frac{\delta_{f_{m}}\left(\delta_{m}-\delta_{0_{m}}\right)}{\delta_{m}\left(\delta_{f_{m}}-\delta_{0_{m}}\right)}$$

(11) $$\tau =\left(1-d\right)K\delta$$

Here, $\delta_{m}$ is the mixed mode [14] separation of the interface; $\delta_{0_{sh}}$, $\delta_{0_{n}}$ is the critical opening between Mode II and Mode I; $G_{\mathrm{shear}}$, $G_{T}$ is the ratio of the strain energy release rate in the shear direction to the total energy release rate; $\delta_{0_{m}}$ represents critical mixed mode [14] separation; $\tau_{0_{m}}$ is the critical tractive force; $\delta_{f_{m}}$ is the final blend mode opening; mixed-mode fracture toughness is $G_{c_{m}}$. B represents the coefficient in the fatigue-crack-growth law, K is the initial stiffness of the material, and d is the exponent of the fatigue-crack-growth law.

BK model definition: Composites exhibit brittle properties to avoid unrealistic material healing in the model, so the following two equations are used for this correction. The value of $K_{sh}$ refers to the stiffness of the penalty for shear, while $\tau_{0_{sh}}$ represents the shear strength of the material.

(12) $$\tau_{0_{sh}}=\tau_{0_{n}}\sqrt{\frac{G_{c_{II}}}{G_{c_{III}}}}$$

(13) $$K_{sh}=K_{n}\frac{G_{c_{I}}}{G_{c_{I}}}\frac{\tau_{0_{sh}}^{2}}{\tau_{0_{n}}^{2}}$$

The above-mentioned theoretical foundation is applied to the cohesive zone model (CZM) for modeling the mixed loading of temperature and stress [27,28]. In the processing of interior components, temperature and pressure are typically interrelated. Changes in temperature may lead to the expansion or contraction of materials, while variations in pressure can impact the structure and stability of interior components. Therefore, understanding the interactive effects between the two is crucial to ensuring the stability of interior components under different operating conditions.

#### 2.3.3. Assumptions of the Model

Since the research model is only one part of the local three-dimensional model of the car dashboard, the edge of the research model that was in contact with the local three-dimensional model of the dashboard was simplified, with its boundary position fixed and no heat exchange occurring with the outside world. In addition, the thicknesses of the upper and lower layers were 1.2 mm and 3 mm, respectively, and the adhesive layer thickness was 0.1 mm. The adhesive layer was partitioned using sweeping technology and cohesive elements. Since the thickness of the adhesive layer is only about 1/10 of the thickness of the upper and lower layers, to ensure simulation accuracy, the adhesive layer mesh density was higher, and the number of elements was greater than that of the upper and lower layers.

While this simplification may affect the simulation results of the temperature field, it was deemed acceptable given the focus on the local area, especially when the impact of the edges on the overall study is minimal. Simplification of the upper and lower layer thickness was conducted to reduce the computational complexity while retaining sufficient accuracy. A higher mesh density was employed for the adhesive layer, to ensure a more accurate simulation of this thin layer.

### 2.4. Model Setup

#### Simulation Parameters

To ensure the simulation’s authenticity, the model’s dimensions and shape were kept consistent with automotive interior components. The material parameters for each model part were assigned according to Table 1 and Table 2 for the polyurethane skin, adhesive, and mesh fabric, respectively. The experiment and subsequent simulation assumed that the properties of the material do not change with temperature changes. Consequently, the bottom surface of the model was fully fixed, and the model was subjected to the same temperature parameter of 70 °C as in the experiment. Pressure was also applied to the model’s top surface, with the parameter set at 0.1 MPa. The entire stamping process was simulated for 10 s. The chosen stamping process parameters aimed to maintain consistency between the model and the actual stamping process, ensuring the model’s effectiveness.

### 2.5. Interlayer Bonding Mechanism

This paper’s research object was the car dashboard’s local adhesive structure, which has a three-layer material composition. The dosage of the curing agent was calculated according to the following ratio.

The reactions that occur during the mixing process are as follows.

(14) $$\begin{array}{c} (Mass\;of\;amine\;curing\;agent\;required\;for\;100\;parts\;by\;weight\;of\;resin)/\%\\ \;\;\;\;\;=\frac{\mathrm{Molecular}\;\mathrm{mass}\;\mathrm{of}\;\mathrm{the}\;\mathrm{amine}}{\mathrm{Number}\;\mathrm{of}\;\mathrm{active}\;\mathrm{hydrogen}\;\mathrm{atoms}\;\mathrm{in}\;\mathrm{the}\;\mathrm{amine}\;\mathrm{molecule}}\times \frac{\mathrm{Weight}\;\mathrm{percentage}\;\mathrm{of}\;\mathrm{epoxy}}{\mathrm{Molecular}\;\mathrm{mass}\;\mathrm{of}\;\mathrm{the}\;\mathrm{epoxy}\;\mathrm{base}} \end{array}$$

(15)
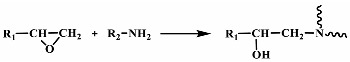


Among them, R, R_1_, R_2_, and R_3_ represent the organic groups, and *n* represents the carbon chain length in the epoxy group. The epoxy group of the epoxy resin undergoes a ring-opening reaction with amine-curing agents to generate isocyanates.

The adhesive that is formed by blending the epoxy resin and curing agent is bonded to the polyurethane skin. The isocyanate reacts with the amine group of the polyurethane skin to form a cross-linked structure, which causes the polyurethane skin to bond with the epoxy resin adhesive [29,30,31].
(16)
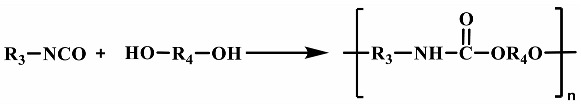


### 2.6. Ultrasonic Assisted Processing

In order to enhance the efficiency and quality of automotive interior component processing, this study introduces an advanced numerical simulation ultrasonic-assisted technique. Diverging from conventional mechanical vibration methods, the numerical simulation ultrasonic-assisted technique employed in this research achieves precise control over the materials by adjusting the temperature and pressure application frequency. This innovative approach provides more flexible operational means and demonstrates significant improvement effects in simulations. The following sections will explain the numerical simulation ultrasonic application curve design utilized in this project and detail its application during the processing.

The applied ultrasonic assistance follows the equation:

(17) $$\alpha =A_{0}+\sum_{n=1}^{N}\left[A_{n}cosn\omega \left(t-t_{0}\right)+B_{n}sinn\omega \left(t-t_{0}\right)\right]$$

where N is the number of Fourier series terms; ω is the angular frequency, measured in rad/s; $t_{0}$ is the initial time; $A_{0}$ is the initial amplitude; $A_{n}$ is the coefficient of the cosine term; $B_{n}$ is the coefficient of the sine term, (*n* = 1, 2, 3, …, N).

## 3. Experimental Study and Investigation of Temperature–Stress Hybrid Loading

### 3.1. Experimental Research on Automotive Interior Component Encapsulation

This study focuses on the localized bonding structure of the automotive dashboard (as shown in Figure 1d), comprising three layers: the top layer (PU leather), the middle layer (Araldite^®^ 2015 two-component epoxy resin adhesive), and the bottom layer (3-D mesh fabric).

#### 3.1.1. Material Composition and Manufacturing Process

**Figure 4:**
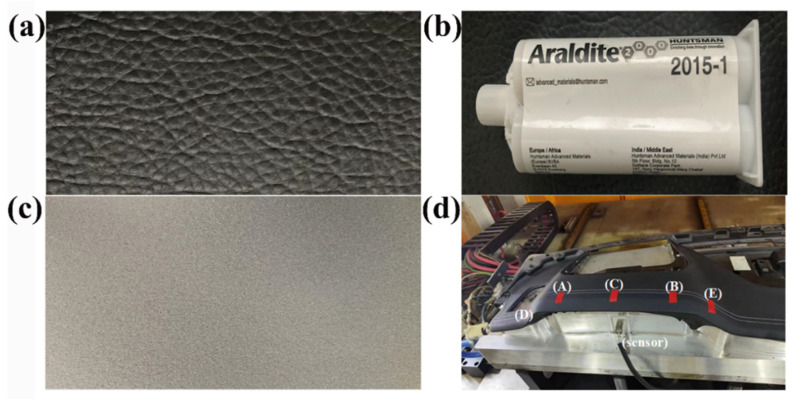
(**a**) Surface skin; (**b**) the glue used in the study, Araldite^®^ 2015; (**c**) non-woven fabric; (**d**) the product and surface zoning.

Figure 4 illustrates the components used on the surface of the automotive dashboard, including the PU leather surface (Figure 4a), Araldite^®^ 2015 epoxy resin adhesive (Figure 4b), and the non-woven fabric in the cushion layer (Figure 4c). The manufacturing process involves the hot pressing of automotive interior components and bonding between the layers, forming a three-layer composite material. Physical image of surface zoning for heating and pressurizing the surface of interior components (Figure 4d).

#### 3.1.2. Automated Encapsulation Technology

Automated encapsulation employs an efficient and cost-effective production process. The 3-D mesh fabric is coated with adhesive, cured, and placed into the lower mold of the device. The hot air supplied through the thermal air duct in Figure 2b activates the adhesive. The PU leather is then placed in the upper mold of the device. Once the adhesive layer is activated, the compression molding process is initiated. Figure 2 depicts the automated encapsulation device (Figure 2a), the thermal air duct (Figure 2b), the workpiece (Figure 2c), and the workpiece in a mold-clamping device (Figure 2d).

#### 3.1.3. Challenges in Automated Encapsulation

While automated encapsulation improves efficiency and reduces costs, challenges arise in complex structures, leading to stress concentration and uneven strain distribution. Effective process monitoring is crucial to ensure consistent quality.

#### 3.1.4. Sensor Monitoring in Encapsulation

Sensors in the form of thin films are placed on the adhesive’s surface (Figure 3d). The sensors involved include temperature sensors (Omega—thin-film platinum RTD sensors), stress sensors (Tekscan—FlexiForce A201), strain sensors (HBM—KFU series), and displacement sensors (MTS sensors—Temposonics R-series). The high sampling frequency of the sensors ensures the capture of subtle changes in the various parameters during the experimental process, providing more accurate and detailed data.

#### 3.1.5. Surface Analysis

**Figure 5:**
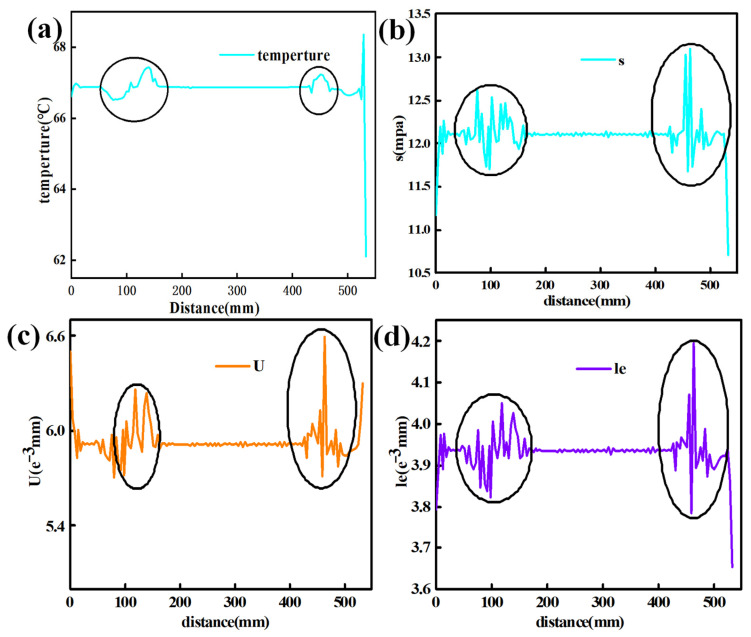
(**a**) Variations in temperature according to position; (**b**) variations in stress according to position; (**c**) variations in displacement according to position; (**d**) variations in strain according to position.

Significant numerical variations in temperature, stress, displacement, and strain on the workpiece’s surface emphasize the necessity of precise control over the surface in the encapsulation and molding of automotive interior components. The temperature, stress, displacement, and strain transmitted to the PC are illustrated in Figure 5.

The area marked with elliptical circles in Figure 5 corresponds to the curved surface area of the product with large curvature on both sides shown in Figure 4d. It can be seen from the areas marked in the legends of Figure 5 that significant numerical changes in temperature, stress, displacement, and strain occur on the curved surface of the product. In automotive interior parts manufactured via wrapping and molding, it is crucial to control defects on the curved surface. In the following sections of this paper, the quality of the automotive interior parts during the processing will be optimized to address the issue of defects on the curved surfaces. By monitoring the production process and analyzing the data, we can better understand the performance changes of the product during processing and make the corresponding adjustments and optimizations, which can improve production efficiency and product quality and will ultimately enhance customer satisfaction.

Temperature variations on the surface can result in the significant thermal expansion and contraction of the adhesive material due to excessive temperature differences, thereby affecting the performance of the bonding layer. In the curved regions of the interior component, stress concentration may lead to structural instability. Substantial displacement and strain can reduce the bonding layer’s strength and the final product’s overall appearance [32]. By integrating the sensor data analysis, manufacturers can enhance production efficiency, improve product quality, and address the challenges associated with automated encapsulation technology.

## 4. Finite Element Analysis

### 4.1. Analysis of the Mixed Loading Effects of Temperature and Pressure

**Figure 6:**
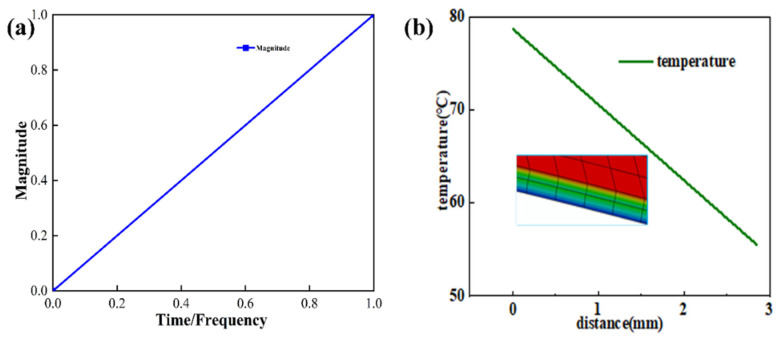
(**a**) Load temperature application amplitude curve; (**b**) temperature transfer diagram from the polyurethane skin to the mesh fabric.

To simulate the temperature and pressure environment during the component wrapping process, pressure was applied to the surface of the polyurethane skin. The temperature was applied as a boundary condition on the upper surface of the top layer of the research model. Since the Araldite^®^ 2015 epoxy adhesive used in the bonding of the composite material has a glass transition temperature of 65 ± 5 °C and an activation temperature of 60 °C, the fully automatic wrapping equipment usually applies pressure for 8–12 s. Since temperature loss occurs through the polyurethane skin, the temperature and pressure applied to the polyurethane skin were 75 °C and 0.1 MPa, respectively, and the loading time was 10 s. Temperature and pressure were applied according to the amplitude curve in Figure 6a.

**Figure 7:**
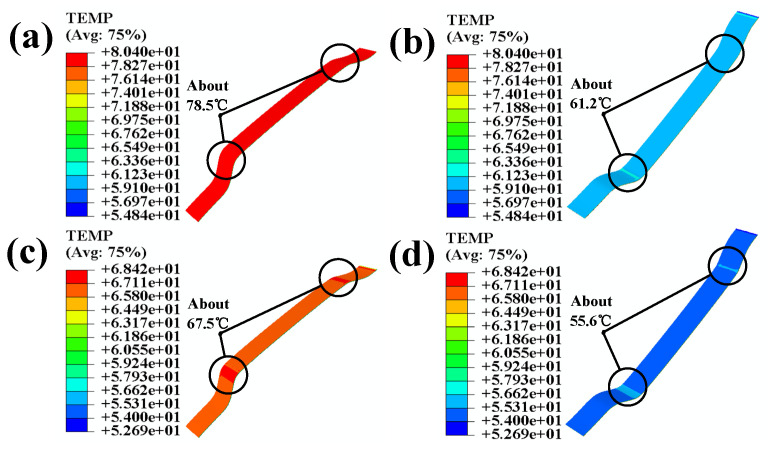
(**a**) Temperature nephogram of the polyurethane skin; (**b**) temperature nephogram of the back of the polyurethane skin; (**c**) temperature nephogram of the adhesive; (**d**) temperature nephogram of back of the 3-D mesh fabric.

Figure 6b illustrates that along the thickness direction (the path from red to blue), at the conclusion of the simulation, the temperature at the adhesive of the interior component reaches the activation temperature of the adhesive. In order to more specifically demonstrate the temperature changes at various levels of automotive interior production after the application of temperature and pressure, we extracted the temperature profiles of each level of the interior components and present them in Figure 7.

After applying temperature and pressure from above the polyurethane skin, the temperature of the polyurethane skin reaches 78.5 °C, as shown in Figure 7a. As it passes through the solid portion of the polyurethane skin and reaches the back side, the temperature reaches the activation temperature of the adhesive, 60 °C; it can be observed that the temperature increases more intensely in areas where the curvature of the polyurethane skin is more significant, as shown in Figure 7b. Once the temperature reaches the activation temperature of the adhesive skin, it begins to bond with the adhesive due to the adhesive’s conductivity; temperature transfer occurs less notably in the direction of the thickness of the interior component. At this point, the temperature above the adhesive rises, especially in those areas with greater curvature of the interior component, as shown in Figure 7c. The temperature reaches 67.5 °C. It can be observed that the temperature increase is more significant in areas with greater curvature compared to that in flat areas. Due to the low thermal conductivity of the adhesive and the pressure that continues to be applied, the temperatures undergo a series of transfers. It decreases to 55.6 °C after reaching the back side of the 3-D mesh fabric. (All “e” in the text is equal to “10” of Scientific notation).

When analyzing the temperature transfer depicted in Figure 7, it is evident that significant temperature variations occur in areas of high curvature in the interior component’s adhesive, according to the experimental results shown in Figure 5; consequently, conducting a more comprehensive study on the combined loading of temperature and stress in regions of high curvature in coated components is necessary. This comprehensive study will help provide a deeper understanding of the effects of temperature and stress on adhesive in areas with large curvatures.

The temperature applied during the simulation was intended to activate the adhesive and its curing temperature, ensuring that the bonding process would mimic real-world conditions. The application of pressure was included to simulate a real processing scenario. In the common processing of interior parts, it is necessary to thermally activate the adhesive and apply a certain level of pressure to promote the bonding of the interior parts. Therefore, the purpose of applying pressure when the upper and lower molds are closed is to work cooperatively with the temperature reaching the curing temperature of the epoxy resin adhesive. This ensures that under a certain pressure, the epoxy resin adhesive bonds with the polyurethane skin. The insights gained from this study will be instrumental in optimizing the design and material choices for components with extensive curved surfaces, ensuring their sustained reliability and functionality over the long term.

**Figure 8:**
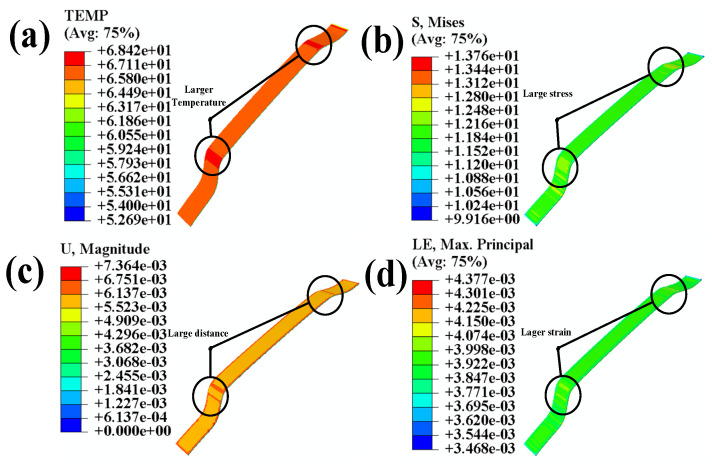
(**a**) Temperature nephogram of adhesion; (**b**) stress nephogram of adhesion; (**c**) displacement nephogram of adhesion; (**d**) strain nephogram of the adhesive.

Figure 8a shows that higher temperature concentrations occur in those areas of adhesion with more significant curvature than the flat regions. Figure 8b reveals the higher stress concentrations in those areas of adhesion with more significant curvature than the flat regions. Figure 8c indicates the significant displacement variations in areas of adhesion curvature. Finally, Figure 8d demonstrates the significant strain in areas of adhesion curvature.

After the combined loading of temperature and stress, the adhesion area undergoes significant concentrations of temperature, stress, displacement, and strain in regions with curved surfaces. These conditions can decrease the adhesive bond quality between the adhesion polyurethane skin and the 3-D mesh fabric [32].

### 4.2. Zonal Optimization Simulation

During the wrapping process of the composite laminated panel, the temperature is the main factor affecting the concentrations of stress and strain in the component, especially in the case of non-uniform areas such as curved surfaces, sharp corners, and edges in the material model. Therefore, controlling the temperature in these different areas is crucial. According to the temperature simulation results, stress appears first. It is more concentrated in highly curved areas, and the change in temperature causes a change in the distribution of the stress field. Therefore, it is necessary to appropriately reduce the temperature in the highly curved areas to make the stress field distribution more uniform. Meanwhile, if the temperature difference is too large, it will cause significant internal and residual stresses, making the deformation of the component more severe.

**Figure 9:**
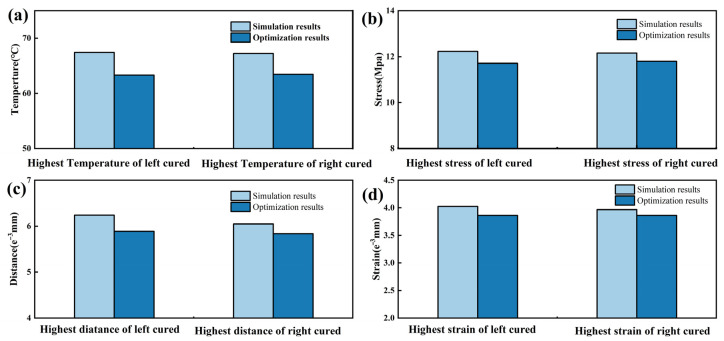
(**a**) Comparison of the temperature values between the direct simulation results and the partitioned simulation results; (**b**) comparison of the stress values between the direct simulation results and the partitioned simulation results; (**c**) comparison of the displacement values between the direct simulation results and the partitioned simulation results; (**d**) comparison of the strain values between the direct simulation results and the partitioned simulation results.

A partitioned simulation approach is deemed advantageous in response to the observed challenges in the adhesive layer within regions of significant curvature. During the partitioned simulation of the model, different boundary conditions were applied to various regions. The applied boundary conditions involved changing the temperature boundary conditions while keeping the rest of the model’s boundary conditions constant. The purpose of applying these boundary conditions is to introduce different temperatures to each segmented area of the polyurethane surface, thereby achieving the application of varying temperatures during the processing of the interior components. Consequently, the model is subdivided into five distinct regions: A, B, C, D, and E. Recognizing the corresponding nature of D and E, as well as of A and B, the model can be effectively grouped into three regions, as illustrated in Figure 1d. Through comprehensive simulations involving the application of temperature, it was determined that the temperature in region C reaches 75 °C, and in regions D and E, it is also 75 °C. Meanwhile, in regions A and B, the temperature is maintained at 70 °C. Temperature loading and pressure are applied based on the amplitudes depicted in Figure 6a, with the pressure on the model remaining constant. Subsequent simulations revealed improvements when compared to the direct application of temperature and pressure to the entire model. A detailed comparison between the partitioned and direct simulation results is presented in Figure 9.

By comparing the simulation results before and after partitioning, we found that after partition heating, the temperature in the highly curved areas decreased but still reached the activation temperature of the adhesive layer. At the same time, the highest temperatures at the two curved surfaces on the left and right sides decreased by 5.61% and 6.13%, respectively. The maximum displacements at the two curved surfaces on the left and right sides decreased by 5.60% and 3.5%, respectively. The stress concentration at the two curved surfaces on the left and right sides decreased by 4.27% and 2.95%, respectively. The maximum strains at the two curved surfaces on the left and right sides decreased by 3.98% and 2.53%, respectively.

By comparing the results of the direct simulation and partitioned simulation, it was observed that partitioning the model surface can reduce the temperature, stress, displacement, and strain in the bonding layer at the curved surfaces to some extent, although the effect is insignificant.

### 4.3. Integrated Ultrasonic Zoning Optimization Simulation

**Figure 10:**
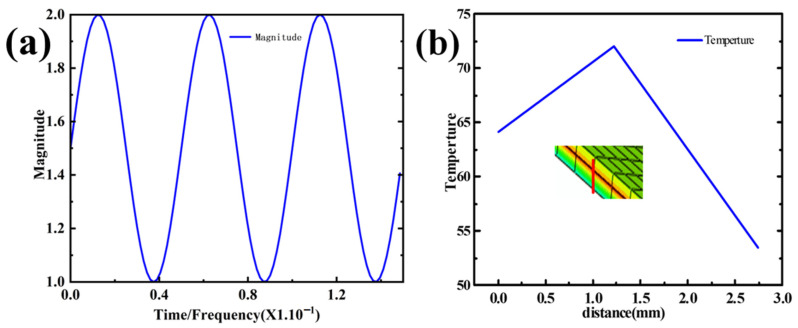
(**a**) Load temperature application amplitude curve; (**b**) diagram of the temperature transfer from the polyurethane skin to the mesh fabric.

Therefore, based on the partitioned simulation of the model, this study proposes a new processing technique using ultrasonic assistance. It was determined that the temperature in region C reaches 55 °C, while in regions D and E, it is also 55 °C. Meanwhile, in regions A and B, the temperature is maintained at 45 °C (partition is shown in Figure 1b). The fitted ultrasonic wave was applied to the utilized pressure and temperature loadings in amplitude, with the circular frequency set at 125,600, the starting time set at 0, and the initial amplitude set at 1.5; in this amplitude set, A and B were each set at 0 and 0.5. The applied ultrasonic assistance followed Equation (17) above; this is shown in Figure 10a. In the article, the temperature ramp-up during the direct simulation and sectional simulation of the interior component is applied according to Figure 6a. Subsequently, in the model’s ultrasonic-assisted processing step, the temperature ramp-up is applied following Figure 10a. In both processes, the objective is to gradually raise the temperature from 25 to 70 °C.

The new processing technique significantly improves the bonding layer’s temperature, stress, displacement, and strain at the curved surfaces. Meanwhile, this technology can reduce the heating temperature; it can change the heating temperature from 75 °C to 55 °C or 45 °C, so it can save a great deal of energy.

**Figure 11:**
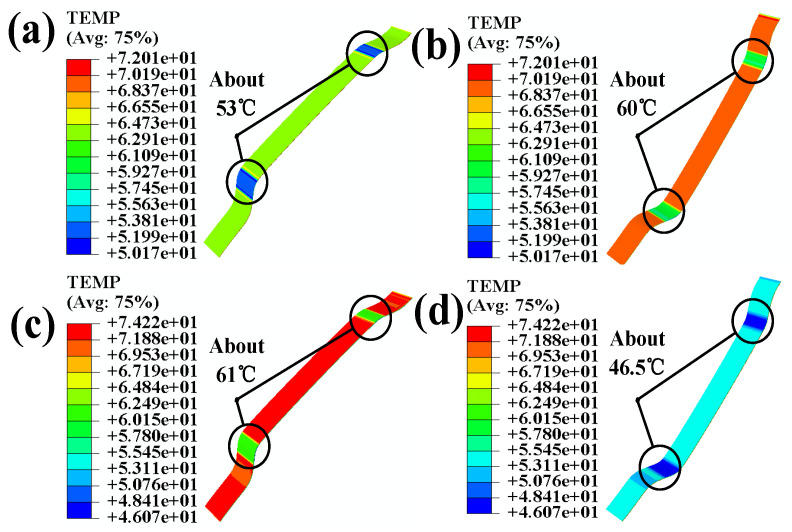
(**a**) Temperature nephogram of the polyurethane skin; (**b**) temperature nephogram of the back of the polyurethane skin; (**c**) temperature nephogram of adhesive; (**d**) temperature nephogram the back of the 3-D mesh fabric.

Figure 10b shows that after simultaneously applying temperature and pressure, the adhesive layer’s temperature remains above the activation temperature of the adhesive. In order to more specifically demonstrate the temperature changes at the various levels of the automotive interior cladding after applying temperature and pressure, we extracted the temperature profiles of each level of the interior components and present them in Figure 11.

In terms of the activation temperature for adhesion during processing, the temperature of the polyurethane skin decreases to 53 °C, the temperature at the back of the polyurethane skin decreases to 60 °C, the temperature at the surface of the adhesive layer decreases to 61 °C, and the temperature decreases to 46.5 °C after passing through the adhesive layer and the 3-D mesh fabric. This represents a significant improvement compared to the scenario where the surface is not divided and the partitioned simulation of the temperature and pressure load are applied, as shown in Figure 6a.

**Figure 12:**
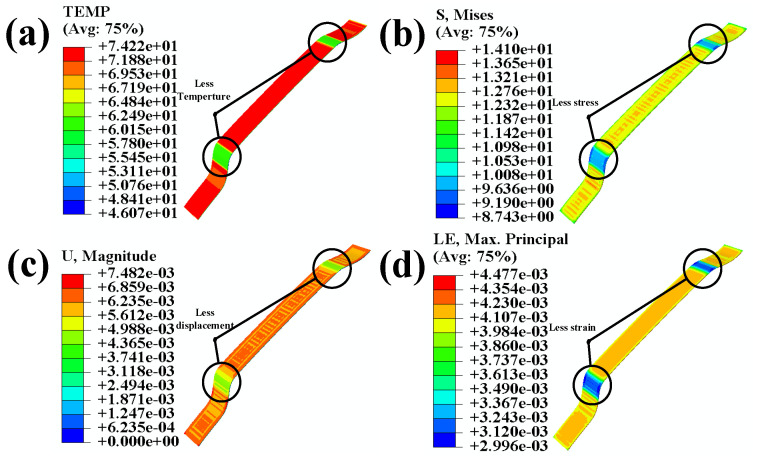
(**a**) Temperature nephogram of the adhesive; (**b**) stress nephogram of the adhesive; (**c**) displacement nephogram of the adhesive; (**d**) strain nephogram of the adhesive.

**Figure 13:**
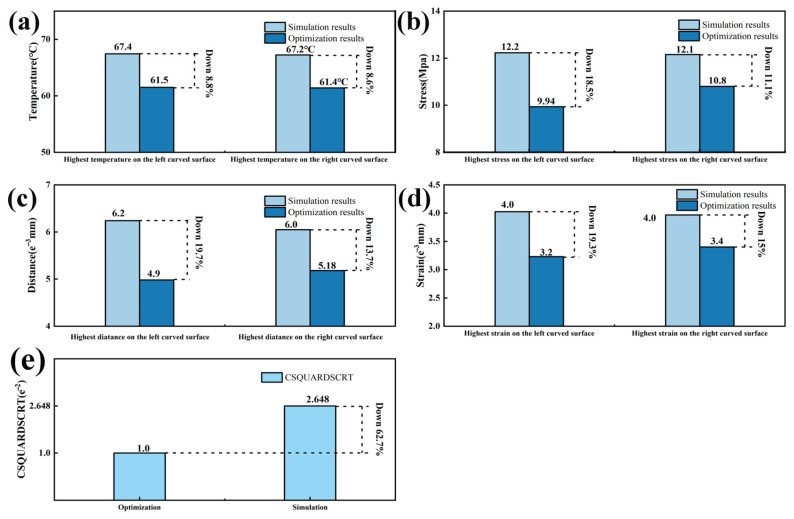
(**a**) Comparison of the temperature values between the direct simulation results and the integrated ultrasonic zoning optimization simulation results; (**b**) comparison of the stress values between the direct simulation results and the integrated ultrasonic zoning optimization simulation results; (**c**) comparison of the displacement between the direct simulation results and the integrated ultrasonic zoning optimization simulation results; (**d**) comparison of the strain values between the direct simulation results and the integrated ultrasonic zoning optimization simulation results; (**e**) changes in adhesive damage.

By observing Figure 8 and Figure 12, it can be seen that there is a significant decrease in temperature, stress, displacement, and strain at the curved surfaces of the automotive interior component. To better demonstrate the significant improvements in automotive interior component manufacturing achieved through the integrated ultrasonic zoning optimization simulation, this study compares the results of the direct manufacturing simulation and the integrated ultrasonic zoning optimization simulation shown in Figure 13.

**Figure 14:**
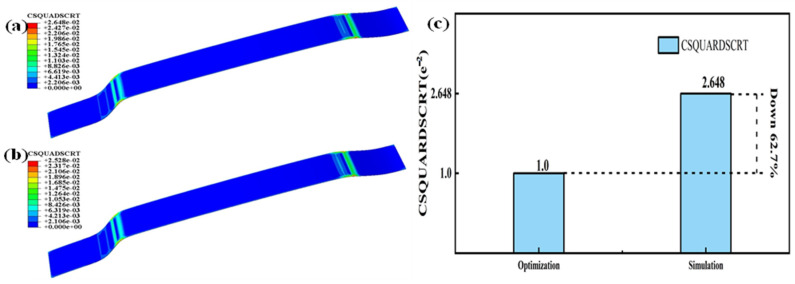
(**a**) Damage nephogram of the adhesive layer without partitioning; (**b**) damage nephogram after the integrated ultrasonic zoning optimization simulation; (**c**) comparison of the damage shown between the direct simulation results and the integrated ultrasonic zoning optimization simulation.

By comparing the simulation results before and after partitioning, it was found that after partition heating, the temperature in highly curved areas decreased but still reached the activation temperature of the adhesive layer. By comparing the results with Figure 9, it can be observed that the integrated ultrasonic zoning optimization simulation has significantly improved in terms of addressing the issues encountered while processing the large curved surfaces of automotive interior components, compared to a direct simulation and a zoning optimization simulation. Compared to the direct simulation results with mixed temperatures and stress loading, the integrated ultrasonic zoning optimization simulation decreased by 8.8% and 8.6% in terms of the highest temperatures at the two curved surfaces on the left and right sides, respectively. The maximum displacements at the two curved surfaces on the left and right sides decreased by 18.5% and 11.1%, respectively. The stress concentration at the two curved surfaces on the left and right sides decreased by 19.7% and 13.7%, respectively. The maximum strains at the two curved surfaces on the left and right sides decreased by 19.3% and 15.0%, respectively. As shown in Figure 14c, the maximum damage of the curved surface decreased by 62.5%.

By observing the damage to the adhesive, it can be noted that concerning adhesive subjected to combined temperature and pressure loading, after surface partitioning and ultrasonic-assisted optimization on the interior component’s surface, the adhesive’s damage decreased by 62.7%. This validates the improvement seen in the adhesive’s damage condition after surface partitioning and the ultrasonic-assisted optimization of the interior component.

## 5. Experimental Verification

**Figure 15:**
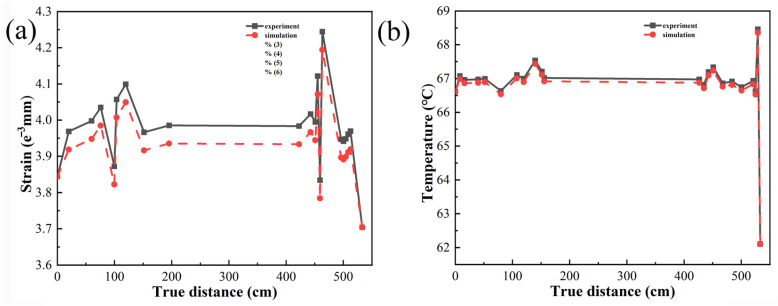
(**a**) Experimental and finite element comparison of strain; (**b**) experimental and finite element comparison of temperature.

These experiments have revealed the occurrence of significant temperature, stress, displacement, and strain in the bonding layer of automotive interior components with curved surfaces (as shown in Figure 5). Additionally, finite element simulations conducted under mixed loading conditions on the automotive interior components have shown that significant differences in temperature, stress, displacement, and strain occur in those areas of the bonding layer with large curvatures. The experimental results and finite element simulations demonstrate a high degree of correlation, and a comparison between the results has been conducted. The comparisons of strain and temperature are depicted in Figure 15.

The findings highlight the consistency between the experimental and numerical approaches, validating the finite element simulations’ accuracy and reliability in capturing the bonding layer’s complex behavior under mixed loading conditions [25,26]. This comprehensive analysis provides valuable insights into the performance and behavior of the adhesive system in automotive interior components with curved surfaces. Such information can be utilized for further optimization and improvement of the design, material selection, and manufacturing processes to enhance the overall quality and durability of the bonded structures.

## 6. Conclusions

Our experimental results reveal pronounced temperature and stress concentration phenomena, accompanied by substantial displacement and strain, in highly curved regions during the processing of automotive interior parts, in contrast to areas with a smaller curvature. Finite element analysis corroborates these findings, highlighting significant changes in temperature concentration, stress, displacement, and strain in highly curved areas compared to regions with milder curvatures. The congruence between experimental and numerical approaches attests to the finite element simulations’ accuracy and reliability in capturing the bonding layer’s intricate behavior under mixed loading conditions.

Following an integrated ultrasonic zoning optimization simulation, temperature, stress, displacement, and strain improvements were observed in the high-curvature regions of automotive interior components during the simulated manufacturing process, and, in comparison to the zoning simulation and direct simulation on polyurethane, the highest temperatures on the two curved surfaces, both left and right, decreased by 8.8% and 8.6%, respectively. The left and right displacements on the two curved surfaces decreased by 18.5% and 11.1%, respectively. The stress concentration on the two curved surfaces, both left and right, decreased by 19.7% and 13.7%, respectively. The maximum strains on the two curved surfaces, both left and right, decreased by 19.3% and 15.0%, respectively. The maximum damage at the curved surface decreased by 62.5%.

Through integrated ultrasonic zoning optimization simulation, the heating temperature can be changed from 75 °C to 55 °C or 45 °C, which markedly reduces the heating temperature and reduces energy consumption.

## Figures and Tables

**Table 1:** The surface skin’s mesh fabric parameters.

Materials	Density (t/mm^3^)	Modulus of Elasticity (MPa)	Poisson’s Ratio	Thermal Conductivity (w/m·k)	Coefficient of Thermal Expansion (1/k)	Specific Heat Capacity (J/ kg·k)
Surface skin	5.58 × 10^−10^	3057	0.45	0.034	1.8 × 10^−8^	1.38 × 10^6^
Mesh fabric	9 × 10^−11^	4000	0.3	0.04	5.94 × 10^−5^	1.1 × 10^9^

**Table 2:** Adhesive layer parameters.

Materials	Density t/mm^3^	Modulus of Elasticity MPa	Rupture Energy MJ/mm^2^	Shear Modulus MPa	Thermal Conductivity w/m·k	Coefficient of Thermal Expansion 1/k	Specific Heat Capacity J/ kg·k	Glass Transition Temperature (Tg) °C
Glue layer	1.45 × 10^−9^	1850	0.43	560	0.234	8.5 × 10^−5^	5.5 × 10^8^	65 ± 5

## Data Availability

The datasets used or analyzed in the current study are available from the corresponding author upon reasonable request.
